# Integrating core physics and machine learning for improved parameter prediction in boiling water reactor operations

**DOI:** 10.1038/s41598-024-56388-5

**Published:** 2024-03-10

**Authors:** M. R. Oktavian, J. Nistor, J. T. Gruenwald, Y. Xu

**Affiliations:** 1 Blue Wave AI Labs, 1281 Win Hentschel Blvd, West Lafayette, IN 47906 USA; 2 School of Nuclear Engineering, Purdue University, 363 North Grant Street, #5281, West Lafayette, IN 47907 USA; 3 Department of Physics and Astronomy, Purdue University, 525 Northwestern Avenue, West Lafayette, IN 47907 USA

**Keywords:** Nuclear reactor, Neural network, Neutron diffusion, Engineering, Power stations, Nuclear physics, Nuclear energy, Nuclear fusion and fission

## Abstract

This study introduces a novel method for enhancing Boiling Water Reactor (BWR) operation simulations by integrating machine learning (ML) models with conventional simulation techniques. The ML model is trained to identify and correct errors in low-fidelity simulation outputs, traditionally derived from core physics computations. These corrections aim to align the low-fidelity results closely with high-fidelity data. Precise predictions of nuclear reactor parameters like core eigenvalue and power distribution are crucial for efficient fuel management and adherence to technical specifications. Current high-fidelity transport calculations, while accurate, are impractical for real-time predictions due to extensive computational demands. Our approach, therefore, utilizes the standard two-step simulation process-assembly-level lattice physics calculations followed by whole-core nodal diffusion computations-to generate initial results, which are then refined using the ML-based error correction model. The methodology focuses on improving simulation accuracy in regular BWR operations rather than developing a universal ML predictor for reactor physics. By training an advanced neural network model on the difference in high-fidelity and low-fidelity simulations, the model can reduce the nodal power error from low-fidelity simulations to around 1% on average and the core eigenvalue down to under 100 pcm. This result is under the condition of the normal variations of control rod pattern and core flow rate changes in standard BWR operations used in the training and evaluation of the machine learning model. This work suggests a promising approach for achieving more accurate, computationally feasible simulation solutions in nuclear reactor operation and management.

## Introduction

### Background

During power operation, nuclear reactors, especially BWRs, require dynamic and precise control of reactivity in order to maintain safe and efficient operation. Several strategies, including control rod adjustments and core flow rate changes, are employed throughout the reactor’s cycle to regulate reactivity. The goal of reactivity control is to maintain stable operations where reactivity is neutral ($\rho =0$ or $k=1$). The total reactivity balance in a BWR can be expressed as:

1 $$\begin{aligned} \rho =\rho _{CR}+\rho _{FR}+\rho _{FB}+\rho _{DP}=0 \end{aligned}$$

where CR represents the control rods, FR represents the flow rate, FB represents feedback, and DP is the depletion effect^1^.

Due to the complex mechanism of reactivity control and its importance, accurate parameter prediction during BWR operations is crucial. Higher accuracy in predicting important nuclear reactor parameters, such as core eigenvalue (or effective neutron multiplication factor, $k_{eff}$) and power distribution, among others, contributes to more effective fuel planning, safe operation, and compliance with plant technical specifications. High-fidelity neutron transport calculations, although accurate, are not practical for real-time core parameter prediction due to their extensive computational time. As a result, the conventional two-step approach-initially involving either single or multi-assembly transport calculations, followed by a comprehensive core diffusion computation-remains prevalent today^2–4^.

Generalized Perturbation Theory (GPT) has seen advancements in reactor physics but faces challenges in real-time analysis and large-scale core design, primarily due to reduced accuracy for significant system changes and high computational costs for higher-order methods^5,6^. Exact-to-Precision GPT (EpGPT) offers improvements in complex reactor analyses, yet its applications are mainly limited to PWR assembly models^7,8^. The computational demand for larger models, like full-core reactors, remains high. This has led to exploring alternatives, such as Machine Learning, which provide significant accuracy improvements over lower fidelity methods without the need for exact system representations, addressing both speed and accuracy concerns in reactor core simulations.

With this in mind, this work proposed a novel approach to simulating BWR operations using conventional reactor simulation assisted by a machine learning-based correction model. The machine learning (ML) model is trained to predict the error of the low-fidelity (LF) simulation results (which are the traditional core physics approach) and then use the predicted error to further improve the solutions. The corrected solutions should be close to the high-fidelity (HF) data used to train the machine learning model, which comes from prepared high-resolution Monte Carlo simulations. This study is not meant to develop a multi-purpose ML prediction model for reactor physics, but instead as a tool to improve simulation and parameter prediction accuracy in the routine BWR operations.

### Simulations in reactor physics

This conventional method in reactor simulation unfolds in two main phases: the lattice physics calculation, performed on the scale of an assembly, followed by a nodal diffusion calculation across the core. The lattice physics calculation involves the use of high-fidelity transport calculation to solve for energy-dependent, spatially detailed angular flux. The transport calculation solves the so-called Boltzmann Transport Equation^9^ as follows:

2 $$\begin{aligned} \begin{aligned} \vec {\Omega } \cdot \nabla \psi \left( \vec {r},\vec {\Omega },E\right) + \Sigma _t\left( \vec {r},E\right) \psi \left( \vec {r},\vec {\Omega },E\right)&= \frac{\chi (E)}{4\pi k_{eff}}\int _0^{\infty } \nu \Sigma _f\left( \vec {r},E'\right) \int _0^{4\pi }\psi \left( \vec {r},\vec {\Omega '},E'\right) d\Omega 'dE'\\&\quad + \int _0^{\infty }\int _0^{4\pi } \Sigma _s\left( \vec {r},\vec {\Omega '} \cdot \vec {\Omega },E' \rightarrow E\right) \psi \left( \vec {r},\vec {\Omega '},E'\right) d\Omega 'dE' \end{aligned} \end{aligned}$$

where $\vec {r}$, $\vec {\Omega }$, and $E$ represent the space, angle, and energy variables, respectively, $\psi$ is the angular neutron flux, and $\Sigma$ is used for the macroscopic cross section and the subscripts $t$, $f$ and $s$ indicate the total, fission, and scattering, $\chi$ is the normalized fission spectrum, and $k_{eff}$ is the effective neutron multiplication factor.

Spatially homogenized and group-condensed macroscopic cross-sections can be generated from the standard flux-weighted cross-section calculation process in lattice physics calculation^10,11^. These data (also called group constants) are required to run any nodal diffusion calculation.

The next step in the reactor physics simulation is to utilize nodal diffusion equations to generate assembly-wise flux solutions and the whole core eigenvalue (also called *k* or $k_{eff}$). The general form of the time-dependent multigroup diffusion equation is given by the equation below^12^:

3 $$\begin{aligned} \frac{1}{\nu }\frac{\partial \phi _g}{\partial t}-\nabla \cdot D_g \nabla \phi _g + \Sigma _{t,g} \phi _g = \sum _{g'=1}^G \Sigma _{s,g'\rightarrow g}\phi _{g'} + \frac{\chi _{pg}(1-\beta )}{k_{\textrm eff}} \sum _{g'=1}^G \nu \Sigma _{f,g'}\phi _{g'} \end{aligned}$$

where the spatial dependence of each quantity is omitted for brevity, and

- $D_g$ = diffusion coefficient for the energy group *g* (cm)
- $\phi _g$ = neutron scalar flux for the energy group *g* (particles/cm^2^ s)
- $\Sigma _{s,g'\rightarrow g}$ = macroscopic scattering cross section from energy group $g'$ to energy group *g* (cm^-1^)
- $\chi _{pg}$ = prompt fission neutron yield in the energy group *g*
- $k_{\textrm eff}$ = effective neutron multiplication factor (core eigenvalue)
- $\nu \Sigma _{f,g}$ = macroscopic fission neutron production cross section at energy *g* (cm^-1^)

The other approach to solving the neutron transport equation is through stochastic methods, like Monte Carlo methods. Monte Carlo methods, in terms of simulation fidelity, are currently the gold standard for modeling neutrons in nuclear reactors^13^. The methods are based on repeated random sampling to obtain numerical results. Due to the nature of the statistical approach, the accuracy of this method depends on the number of samples (or neutrons) and therefore drives up the computational cost to obtain accurate results^14^. Consequently, real-time, high-resolution Monte Carlo simulations for an entire reactor are not currently viable.

### Deep learning with neural networks

The machine learning model in this work utilizes Deep Neural Networks (DNNs) architecture, especially in the category of Convolutional Neural Networks (CNNs). DNNs are multi-layered structures in artificial neural networks, essential for deep learning and handling complex tasks like classification and regression^15^. A DNN employs layers of neurons, each defined by weights ($\textbf{W}$) and biases ($\textbf{b}$), and utilizes activation functions like sigmoid or ReLU. The network aims to minimize a loss function, such as Mean Squared Error (MSE) in typical regression applications. Training involves backpropagation for updating weights and biases, guided by the gradients computed from the loss function. Despite their efficiency, DNNs are computationally demanding and often criticized for their lack of interpretability.

Convolutional Neural Networks (CNNs) specialize in analyzing visual data^16^. They comprise convolutional, pooling, and fully connected layers. Outside of image and visual applications, CNNs have shown significant utility in various fields, including protein structure prediction^17^ and time series forecasting^18^. Additionally, the encoder-decoder architecture is an important model in deep learning, particularly for tasks like sequence-to-sequence predictions, machine translation, and image captioning. This architecture consists of two main parts: the encoder, which processes the input data and compresses the information into a context vector, and the decoder, which takes this vector to produce the output. In this work, CNNs are utilized to both capture patterns in spatial data of BWR operations and decode the processed data into the regression output.

## Results

### Improvement on neutron multiplication factor

The initial metric discussed in this section is related to the performance of the model in the correction of the core $k_{\textrm eff}$ or eigenvalue for the full core BWR model. Achieving a precise $k_{\textrm eff}$ is crucial as it governs the critical condition of the reactor. Accurate values enable reactor operators to make well-informed decisions regarding fuel management and overall safety.

The model evaluation was executed on a test dataset, comprising 15% of the total dataset, isolated during the initial stages of data preprocessing. This dataset, comprising 360 data points, was not used in any other phase of this study. Therefore, the test data provide an unbiased performance metric for the ML model.

**Figure 1:**
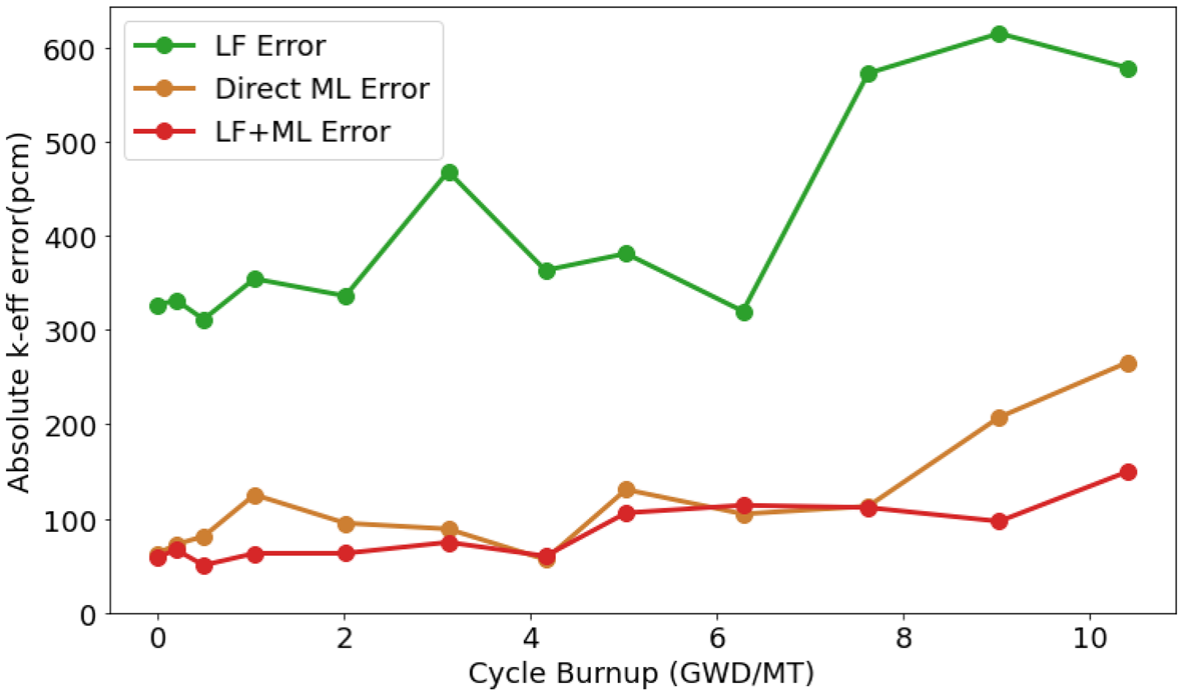
Comparison of averaged $k_{{\textrm eff}}$ error for LF simulation, Direct ML prediction and the proposed approach LF + ML model on the test dataset. Errors are calculated based on absolute discrepancies to high-fidelity data. Note that 1 pcm = $1\times 10^{-5} \Delta k$. Image was generated using Python Matplotlib Library.

As illustrated in Fig. 1, the average $k_{\textrm eff}$ error for all test data in the Hatch-1 Cycle 1 model is presented. The figure includes errors from LF simulation, Direct ML, and our novel approach, LF + ML. Noticeably, the LF errors fluctuate between 300 and 600 pcm. In contrast, both ML methods exhibit substantially lower errors, underlining their superior performance.

The ML-based correction model shows a pronounced improvement in $k_{\textrm eff}$ values, even when compared to the well-validated LF simulator. The high accuracy of direct ML predictions for $k_{\textrm eff}$ is attributed to the fact that $k_{\textrm eff}$ in typical BWR operations is very close to 1.0. This narrow target range enhances predictability. Table 1 reveals that both ML methods produce errors of around 100 pcm in both Root Mean Squared Error (RMSE) or mean absolute error, a considerable reduction from the LF errors. Interestingly, the LF + ML model outperforms Direct ML, especially in terms of the maximum error observed. This can be attributed to the LF + ML approach initiating with a more accurate LF dataset, therefore, avoiding large, nonphysical errors in most scenarios that Direct ML might exhibit. However, even with LF + ML, the predicted $k_{eff}$ is still lack of maximum error improvement due to the diversity in the error distribution between training and test data.

**Table 1:** $k_{{\textrm eff}}$ Errors on test data for all cases.

Methods	RMSE (pcm)	Avg. Err. (pcm)	Max. Err. (pcm)	Std. Dev. (pcm)
LF simulation	460.4	413.2	999.5	162.2
Direct ML prediction	137.8	117.1	826.9	106.4
LF simulation + ML correction	103.1	84.7	485.2	79.1

Note that 1 pcm = $1\times 10^{-5} \Delta k$ for the error term.

### Improvement on nodal power distribution

**Figure 2:**
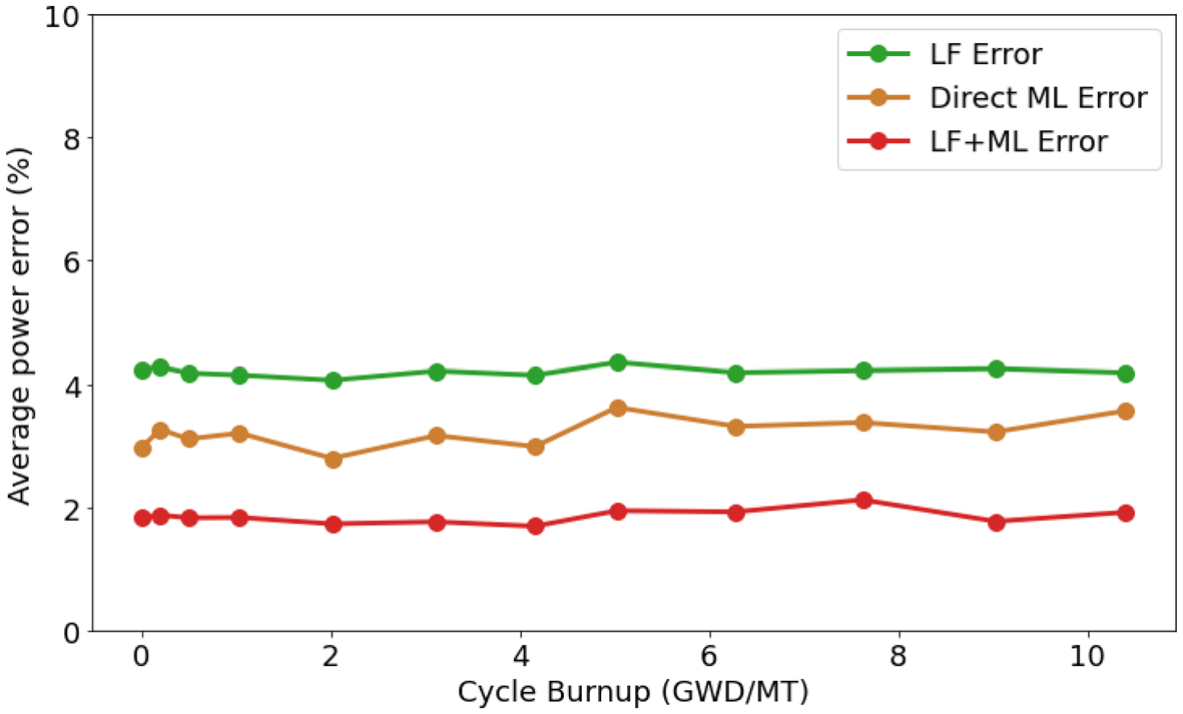
Comparison of averaged nodal power error on the test dataset. Errors are calculated based on the absolute discrepancies to the high-fidelity simulations. Image was generated using Python Matplotlib Library.

The nodal power errors in Fig. 2 explain the advantages of leveraging the ML-based correction model for the diffusion solver. Unlike the previous observations, the Direct ML method performs better than LF but still falls short when compared to LF + ML. The average errors for the prediction of nodal power are around 4.2% for LF simulation and 3.1% for the Direct ML method.

Predicting 3D variables like nodal power is quite a challenge for Direct ML methods. The larger data prediction requirements, coupled with the limitation of available training data, make it difficult to achieve satisfactory performance. This is particularly important given that high-fidelity data collection can be quite expensive, especially for the large-size BWR core.

However, the integration of a low-cost diffusion solver as a starting point for ML models has proven to be beneficial. The LF + ML model has managed to reduce the error to around 1.8% on average, displaying its efficacy even when only a small amount of training data is available. This is important considering that collecting high-fidelity data used as ground truth is resource-intensive.

**Table 2:** Nodal power errors on test dataset.

Methods	RMSE (%)	Avg. Err. (%)	Max. Err. (%)	Std. Dev. (%)
LF simulation	5.9	4.2	34.1	4.6
Direct ML prediction	4.5	3.1	132.0	5.0
LF simulation + ML correction	2.2	1.8	31.8	2.4

The data in Table 2 emphasize that the proposed approach, LF + ML performs substantially better in terms of average error, maximum error, and standard deviation compared to the other methods. This supports the idea for the integration of machine learning techniques with conventional LF approaches to improve the accuracy of nodal power prediction.

**Figure 3:**
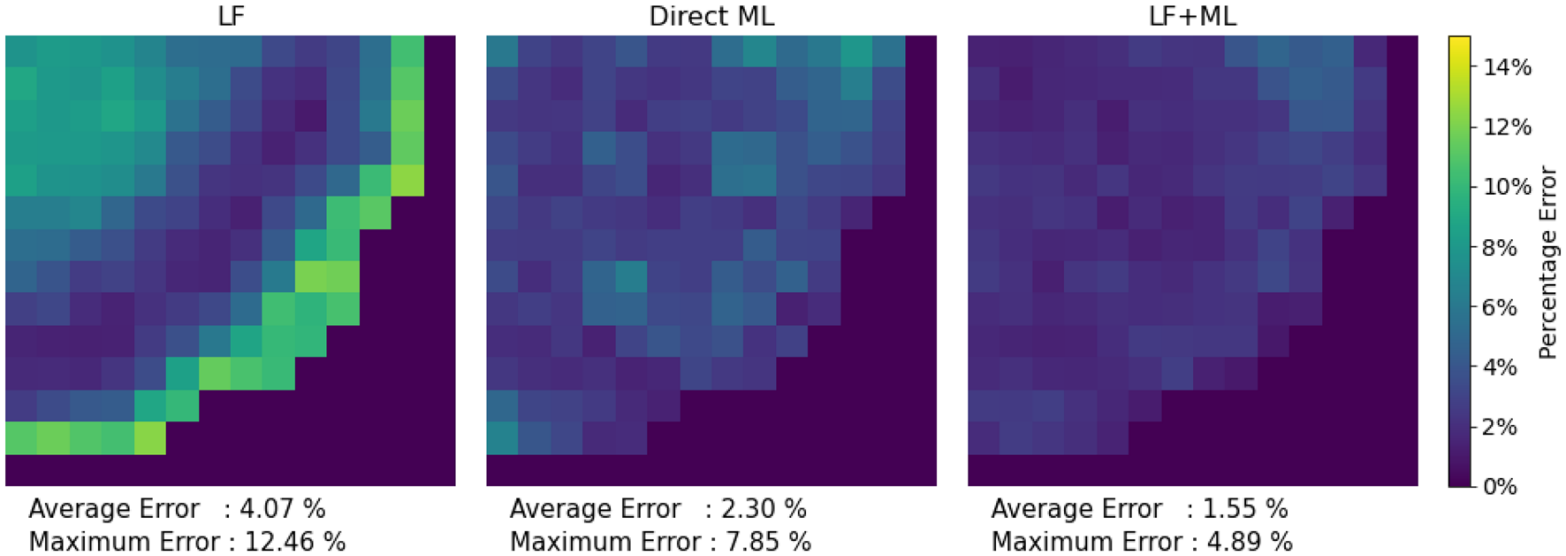
Colormap of the beginning of cycle radial power errors for Hatch-1 Cycle 1 Full core. Images were generated using Python Matplotlib Library.

**Figure 4:**
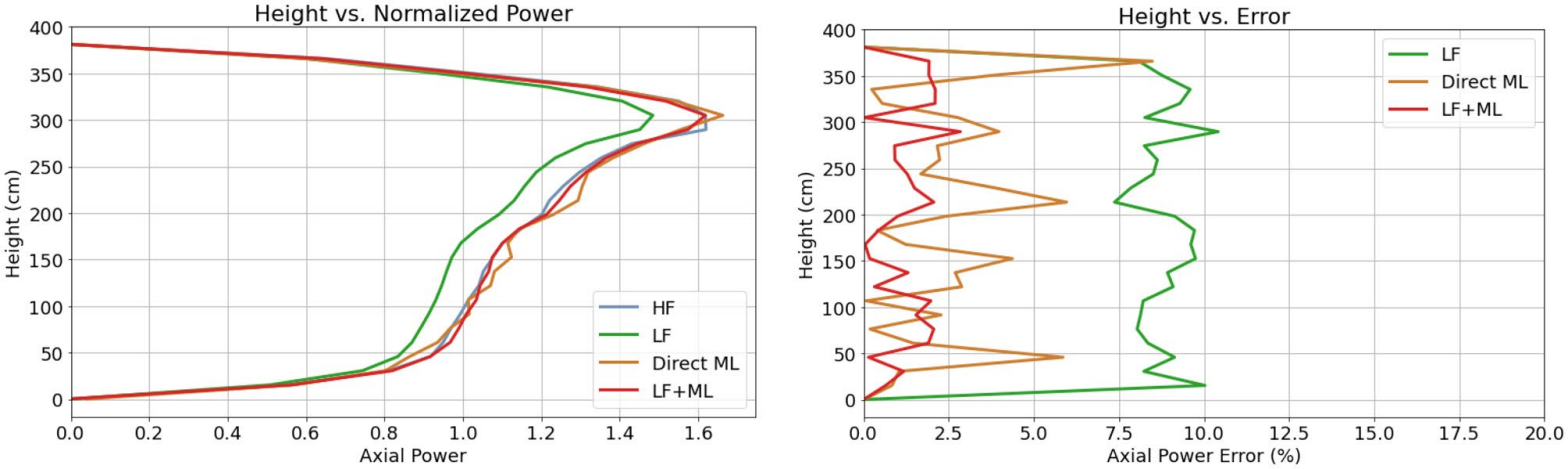
Comparison of the beginning of cycle axial power and errors for the BWR core model. Images were generated using Python Matplotlib Library.

**Figure 5:**
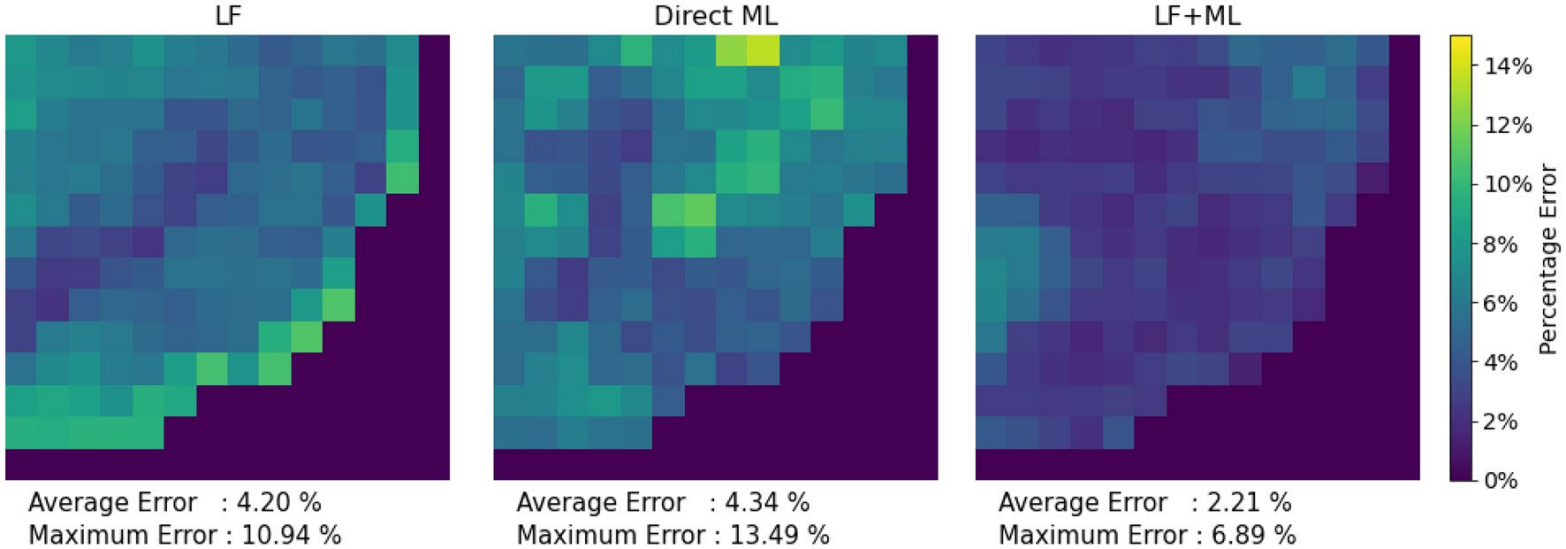
Colormap of the end of cycle radial power errors for the BWR core model. Images were generated using Python Matplotlib Library.

The subsequent plots in Figs. 3, 4 and 5 focus on the radial and axial power distribution for the BWR core model during both the beginning of the cycle (BOC) and end of the cycle (EOC). Generally, at BOC, the material gradients between fuel assemblies create larger errors in diffusion codes. This is particularly noticeable in fuel assemblies containing significant amounts of burnable absorbers.

Figure 3 shows that the LF + ML model outperforms both Direct ML and LF simulation during the BOC. The largest errors are usually localized near the reactor boundary, which is a common challenge in diffusion solver models. In this case, the Direct ML model shows exaggerated errors near the reactor boundary. However, LF + ML utilizes the better initial estimates from LF simulation and refines them, resulting in significantly reduced errors.

Axial power distribution is another critical metric in BWR reactors. The presence of voids in the upper parts of the reactor and the rod insertion in the lower regions of the reactor creates additional challenges to diffusion solvers. Figure 4 reveals that the LF + ML model can effectively correct the errors in the axial power distribution introduced by the LF physics model alone.

In the EOC, LF simulation results still exhibit some errors, especially in the periphery, where there are fuel-reflector interfaces. Figure 5 indicate that the proposed LF + ML model continues to offer superior performance in the power distribution.

### Computational time

Table 3 presents a comparison of compute time for simulating the Hatch-1 BWR case using four different methods: HF, LF, Direct ML, and a hybrid approach of LF + ML. Among these methods, Direct ML stands out for its efficiency, requiring less than 0.1 seconds for this case. On the other hand, HF data collection requires 176 CPUs and takes considerably longer.

**Table 3:** Running time comparison.

Method	Processing units	Running time
HF simulation	176 CPUs	6 hours
LF simulation	1 CPU	3 minutes
Direct ML inference	1 GPU	$<0.1$ seconds
LF simulation + ML inference	1 CPU + 1 GPU	3 minutes

Table 4 outlines the parameters used for simulations in LF and HF data collection. Both methods ran a total of 200 cycles. LF simulation operated on a single CPU, taking a total of 10 hours, which amounts to 10 CPU hours. In contrast, HF data collection required 176 CPUs and had a running time of 50 days, resulting in a total of 211,200 CPU hours. This difference in resource usage and time emphasizes the trade-offs between computational efficiency and simulation fidelity.

**Table 4:** Compute requirements for data collection.

Parameters	Low-fidelity simulation	High-fidelity simulation
Total cycle runs	200	200
Processing units	1 CPU	176 CPUs
Total running time	10 hours	50 days
Total CPU time	10 CPU-hours	211,200 CPU-hours

Collecting accurate HF data poses challenges due to significant computational needs. Even without tight multiphysics coupling, Monte Carlo neutron transport runs still take a considerable amount of resources to finish. In this case, there is a tens of thousands of times difference in the compute resources required for running HF simulations compared to LF simulations.

## Discussion

One of the key observations from the results is the inferior performance of the Direct ML model compared to the LF + ML approach. This difference in performance is attributed to the complexities of full-core reactor simulation, which necessitates a comprehensive grasp of neutron transport described in Eq. 2 as well as thermal hydraulics and material behavior. Being data-driven, Direct ML methods typically fall short of encapsulating the fundamental physics that traditional simulation techniques inherently include.

Reactor physics models often rely on interconnected differential equations to describe neutron behavior, including the diffusion equation in Eq. 3. These equations are solved in conjunction with thermal-hydraulic models to obtain a self-consistent solution for reactor variables such as nodal power distribution and $k_{{\textrm eff}}$. The Direct ML model, as an entirely empirical approach, might overlook the nuances of these interrelated equations. This oversight is apparent in the significant errors noted, particularly near areas with steep material variations or intricate geometries, like the reactor boundaries and control rod locations.

The LF + ML model takes advantage of the initial estimates provided by LF simulations to refine the predictions. This hybrid approach allows for a more physically informed ML model that starts from a reasonable approximation rather than making predictions from random weights. As a result, the LF + ML model effectively leverages the strengths of both paradigms: the physical rigor of traditional simulation methods and the flexibility and computational efficiency of machine learning. This makes it better suited for complex, full-core simulations where understanding the fundamental physics is crucial for accurate and reliable predictions.

In the proposed approach, a possible source of error and uncertainty is the training data. With only 200 cycle runs available, the quantity of data is limited, which can impact the ML model’s ability to generalize and accurately predict reactor behavior. This is because the neural network may not have enough examples to learn the complex relationships between input parameters and reactor variables. As a result, despite the LF + ML model’s superior performance compared to the Direct ML approach, it is not immune to inaccuracies. However, acquiring additional data through more HF simulations comes with significant costs, emphasizing the importance of conducting a cost-benefit analysis.

Future research should concentrate on answering problems regarding cost-benefit analysis, the use of measurement data, and model generalization. Considering the substantial costs associated with acquiring high-fidelity data from Monte Carlo simulations is essential in applying these methods to reactor core design and operations. Ideally, if the actual reactor’s measurement data are available, the data can be easily used as the ground truth for the ML-based correction model. However, such measurement data often contain noise, and it may not be feasible to obtain data for every parameter of interest.

## Methods

### Low-fidelity and high-fidelity data

The LF model was made in the US NRC codes, Purdue Advanced Reactor Core Simulator (PARCS)^19^. This model consists of three different fuel bundles labeled each with varying uranium enrichment and gadolinia concentration. The model includes 560 fuel bundles encircled by reflectors. Along with the radial setup, there are 26 axial planes made up of 24 fuel nodes, plus a node of reflectors at the top and bottom planes.

In this work, the model was made in quarter symmetry to save computational time and further reduce the data complexity^20^. The symmetry was conducted in the radial direction only. The axial discretization was explicitly modeled from bottom to top of the reactor, from reflector to reflector. This is because BWR’s axial variation is not symmetrical axially, so it is required to model it in sufficient detail. Based on this description, the boundary condition was set to be reflective in the west and north of the radial core and vacuum (zero incoming neutron currents) for the other directions.

For developing the ML model, the depletion steps were reduced to 12 steps, from the typical 30–40 depletion steps. The PARCS cross-section library was generated using CASMO-4 for fuel lattices and reflectors. The library includes group constants from eight lattice simulations over control rod positions, coolant density, and fuel temperature. Lattices were simulated at 23 kW/g of heavy metal power density to a burnup of 50 GWd/MT of initial heavy metal.

The HF data were collected using Serpent^21^ Monte Carlo simulations. The model was created to reproduce PARCS solutions on the same core conditions but with higher resolutions and using the state-of-the-art simulation approach. This means no diffusion approximation and continuous energy neutron transport was modeled in detailed geometry structures. Each Serpent calculation was run on 500,000 particles, 500 active cycles, and 100 inactive cycles. The other simulation settings were also optimized for depletion calculations.

### Reactor model

The reactor model used in this work is based on cycle 1 of the Edwin Hatch Unit 1 nuclear power plant. The power plant, located near Baxley, Georgia, is a boiling water reactor of the BWR-4 design, developed by General Electric, with a net electrical output of approximately 876 MWe and 2436 MWth of thermal output. Since its commissioning in 1975, Unit 1 has operated with a core design containing uranium dioxide fuel assemblies, utilizing a direct cycle where water boils within the reactor vessel to generate steam that drives turbines.

The specification of cycle 1 of Hatch reactor unit 1 is presented in Table 5. While it is a commercial, large power plant, Hatch 1 is not as large as a typical 1,000 GWe LWR. Some BWR designs also have about 700-800 assemblies. Nevertheless, due to the availability of the core design for this work, it is generally viable to use this model as a test case.

**Table 5:** Edwin Hatch Unit 1, cycle 1 specifications.

Specification	Value
Core size	26 $\times$ 26
Core diameter	4.27 meters
Core height	3.96 meters
Number of fuel bundles	560
Number of control blades	137
Thermal power	2436 MWth
Electric power	876 MWe

**Figure 6:**
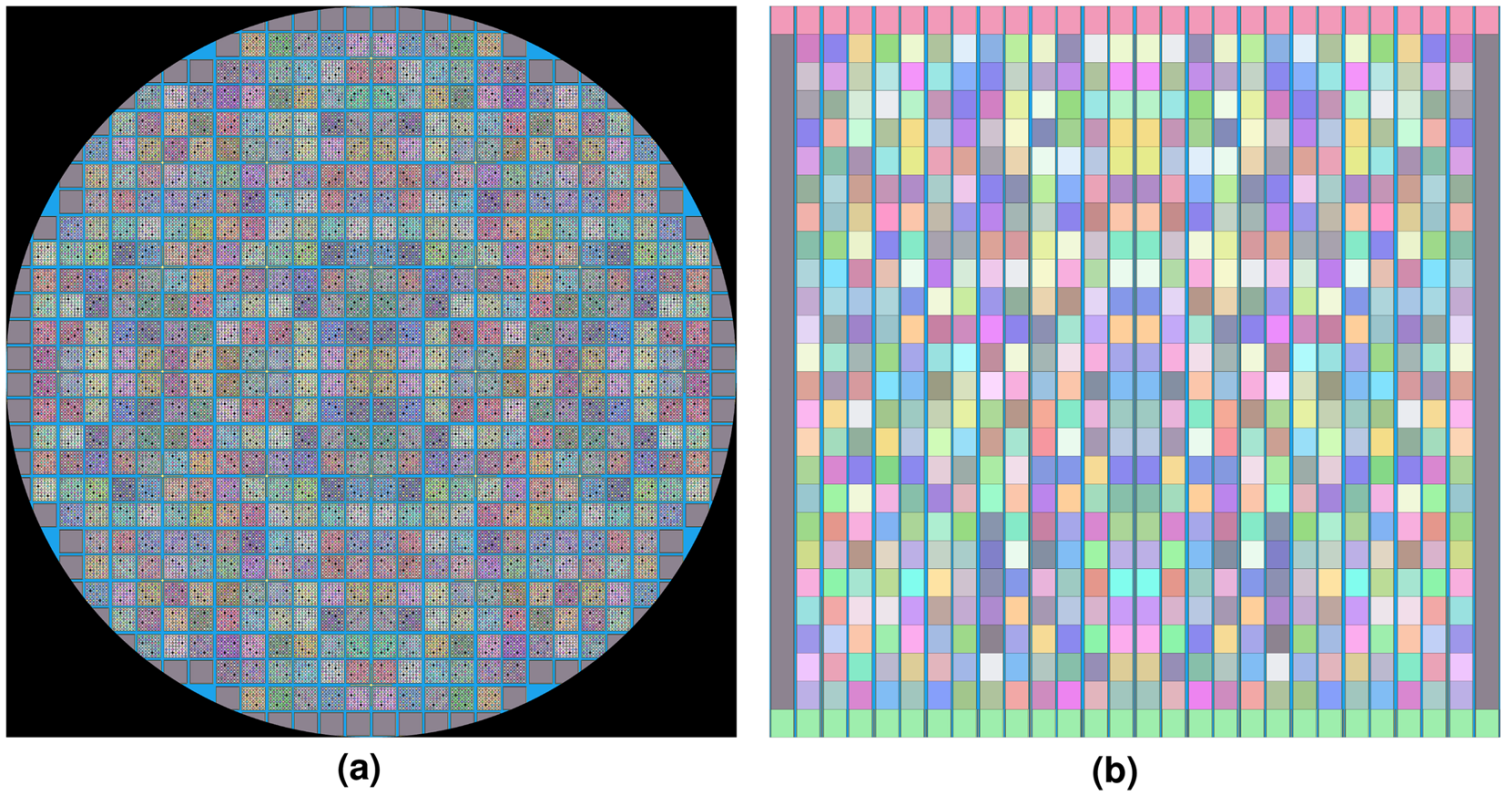
Geometry representation of the full-size BWR core modeled in Serpent. Images were generated by the Serpent geometry plotter.

There are 560 fuel bundles the size of a 7 $\times$ 7 GE lattice in the Hatch 1 Cycle 1 model. Out of the number of fuel bundles in the cycle 1 core, there are three different types of fuels with varying enrichments and burnable absorbers. Using the procedures in running the Serpent model, high-resolution simulations were obtained as shown in the geometry representation in Fig. 6. In the figure, different colors represent different material definitions in Serpent. Because of how the materials were defined individually, the color scheme shown also varied from pin to pin and assembly to assembly. The individual material definition in the pin level was required to capture the isotopic concentration and instantaneous state variables at different fuel exposures and core conditions.

### Data processing

There are 2400 data points collected as samples for this work with various combinations of control blade patterns and core flow rates and 12 different burnup steps. These data points are translated from 200 independent cycle runs for both PARCS and Serpent to provide LF and HF simulation data, respectively. The collected data were processed into a single HDF5 file.

The data processing parts are performed through data split procedures and data normalization. The data is separated into different sets, with a training-validation-test ratio of 70:15:15. The training data is used to teach the network, the validation data to tune hyperparameters and prevent overfitting, and the test data to evaluate the model’s generalization performance on unseen data. From the 2400 data points (200 cycles), the dataset was separated into:

1. Train Dataset: 140 runs or 1680 data points
2. Validation Dataset: 30 runs or 360 data points
3. Test Dataset: 30 runs or 360 data points

**Figure 7:**
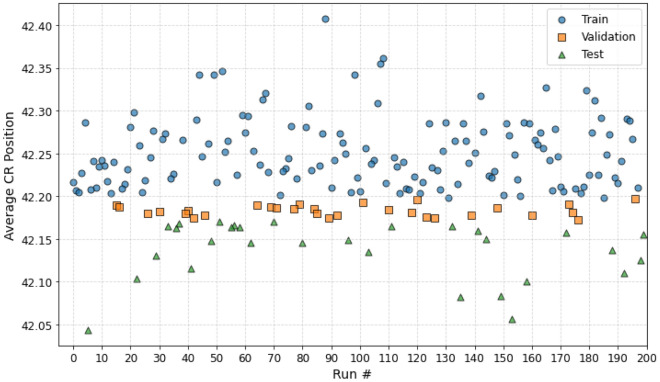
Train-validation-test data split based on average control blade position in the BWR core. Image was generated using Python Matplotlib Library.

The data splitting process was not conducted randomly, but based on the average control blade position in a cycle run. Figure 7 presents the distribution of the average control rod inserted in the reactor. The maximum number of steps is 48 for fully withdrawn blades. In the plot, it can be inferred that the test data have the lowest average CR position (largest insertion), followed by the validation set, and the train data have the highest average CR position (smallest insertion).

The CR-based splitting for the dataset has the purpose of demonstrating the generalization of the model on out-of-sample CR position data. On the other hand, random splitting is not preferred for small datasets, like this problem as the ML model tends to overfit (or imitate) the data. The fixed (CR-based) splitting process used here ensures that the model can perform well on data with a different distribution than the training dataset.

After splitting the data, normalization of the data is important for the ML model to ensure data integrity and avoid anomalies. In this context, the data processing employs Min-Max scaling, a common normalization technique, to rescale the features to a range [0, 1]. This is achieved by subtracting the minimum value of each feature and then dividing by the range of that feature. The scaling is conducted to fit the training data using the MinMaxScaler class from the scikit-learn package then apply the same scaling to the validation and testing data.

### Machine learning model

The target parameters used here are the core eigenvalue (or $k_{\textrm eff}$) and power distribution. The ML model will provide the correction (via predicted errors) of the target parameters that can be used to obtain the predicted HF parameters of interest. The perturbed variables are the parameters that are varied and govern the data collection process and in ML modeling. In this case, the perturbed variables are summarized in Table 6.

**Table 6:** List of perturbed variables for BWR core model.

Perturbed variables	Data shape	Value range
Control blade position	1D or 2D array of integers	0–48 steps
Core flow	Scalar (continues)	40–100%

In this work, a neural network architecture, called BWR-ComodoNet (Boiling Water Reactor—Correction Model for Diffusion Solver—Network) is built which is based on the 3D–2D convolutional neural network (CNN) architecture. This means that the spatial data in the input and output are processed according to their actual dimensions, which are 3D and 2D arrays. The scalar data are still processed using standard dense layers of neural networks.

**Figure 8:**
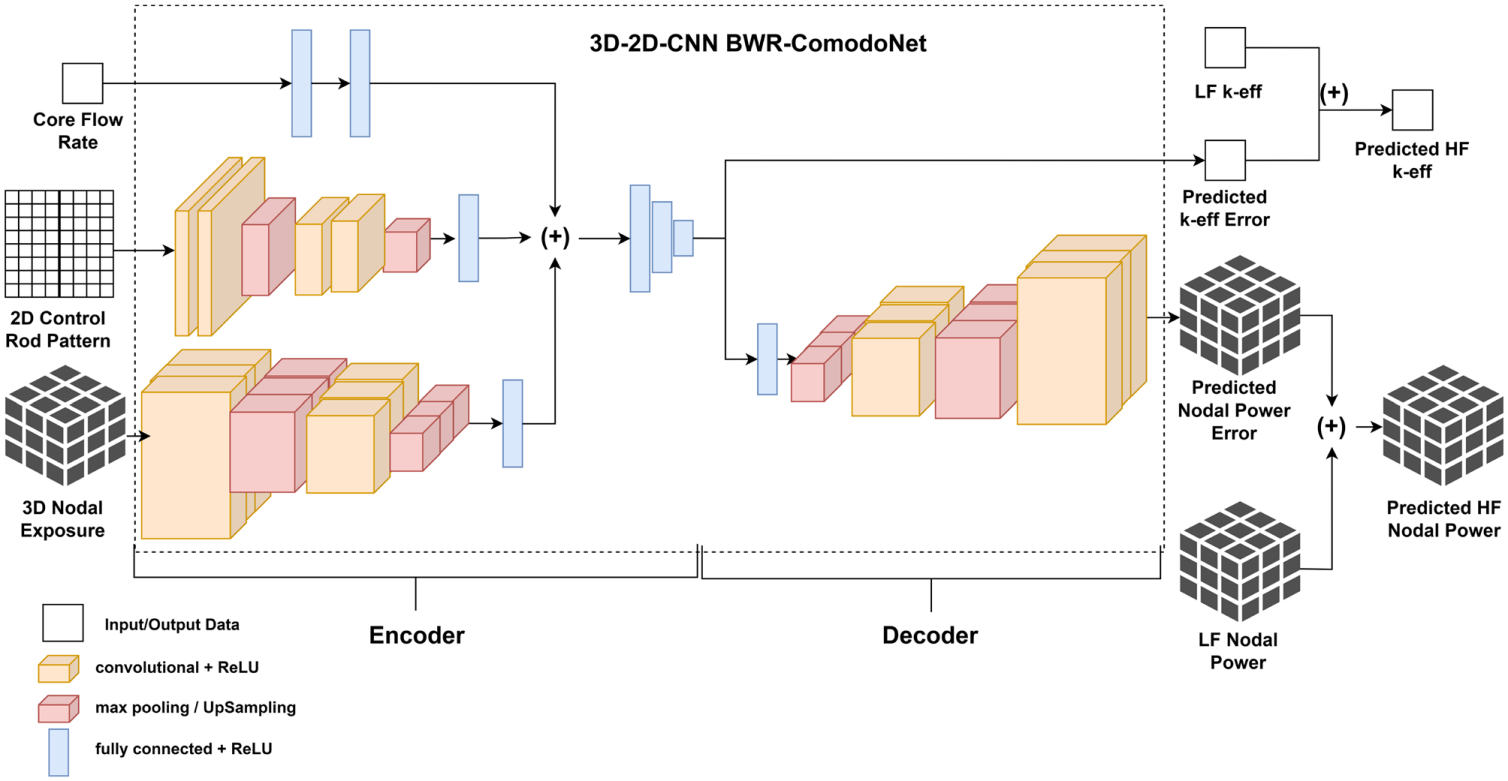
Architecture of BWR-ComodoNet using 3D-2D CNN-based encoder-decoder neural networks. Image was generated using draw.io diagram application.

The architecture of the BWR-ComodoNet is presented in Fig. 8. The three input features: core flow rate, control rod pattern, and nodal exposure enter three different channels of the network. The scalar parameter goes directly into the dense layer in the encoding process, while the 2D and 3D parameters enter the 2D and 3D CNN layers, respectively. The encoding processes end in the step where all channels are concatenated into one array and connected to dense layers.

The decoding process follows the shape of the target data. In this case, the output will be both $k_{\textrm eff}$ error (scalar) and the 3D nodal power error. Since the quarter symmetry is used in the calculation, the 3D nodal power has the shape of (14,14,26) in the x,y, and z dimensions, respectively. BWR-ComodoNet outputs the predicted errors, so there is an additional post-processing step to add the LF data with the predicted error to obtain the predicted HF data.

The output parameters from the neural network model comprise errors in the effective neutron multiplication factor, $k_{eff}$, and the errors in nodal power, which is quantified as:

4 $$\begin{aligned} \begin{array}{l} e_{k} = k_H-k_L \\ \vec {e}_{P} = \vec {P}_H-\vec {P}_L \end{array} \end{aligned}$$

Here, $e_k$ denotes the error in $k_{eff}$ and $\vec {e}_{P}$ represents the nodal power error vector. The subscripts *H* and *L* indicate high-fidelity and low-fidelity data, respectively. According to the equation, the predicted high-fidelity data can be determined by adding the error predictions from the machine learning model to the low-fidelity solutions^22^.

Given the predicted errors, $\hat{e}_k$ and $\hat{\vec {e}}_{P}$, the predicted high-fidelity data, $k_H$ and $\vec {P}_H$ is defined as:

5 $$\begin{aligned} \begin{array}{l} k_H = k_L + \hat{e}_k = k_L + \mathscr {N}_k(\varvec{\theta }, \textbf{x}) \\ \vec {P}_H = \vec {P}_L + \hat{\vec {e}}_{P} = \vec {P}_L + \mathscr {N}_P(\varvec{\theta }, \textbf{x}) \end{array} \end{aligned}$$

where $\mathscr {N}_k(\varvec{\theta }, \textbf{x})$ and $\mathscr {N}_P(\varvec{\theta }, \textbf{x})$ are the neural networks for $k_{eff}$ and power with optimized weights $\varvec{\theta }$ and input features $\textbf{x}$. Although Eq. 5 appears to represent a linear combination of low-fidelity parameters and predicted errors, it is important to note that the neural network responsible for predicting the errors is inherently non-linear. As a result, the predicted error is expected to encapsulate the non-linear discrepancies between the low-fidelity and high-fidelity data.

The machine learning architecture for predicting reactor parameters is constructed using the TensorFlow Python library. The optimization of the model is performed through Bayesian Optimization, a technique that models the objective function, which in this case is to minimize validation loss, using a Gaussian Process (GP). This surrogate model is then used to efficiently optimize the function^23^. Hyperparameter tuning was conducted over 500 trials to determine the optimal configuration, including the number of layers and nodes, dropout values, and learning rates.

The activation function employed for all layers is the Rectified Linear Unit (ReLU), chosen for its effectiveness in introducing non-linearity without significant computational cost. The output layer utilizes a linear activation function to directly predict the target data.

Regularization is implemented through dropout layers to prevent overfitting and improve model generalizability. Additionally, early stopping is employed with a patience of 96 epochs, based on monitoring validation loss, to halt training if no improvement is observed. A learning rate schedule is also applied, reducing the learning rate by a factor of 0.1 every 100 epochs, starting with an initial rate. The training process is conducted with a maximum of 512 epochs and a batch size of 64, allowing for sufficient iterations to optimize the model while managing computational resources.

It is important to note that the direct ML model mentioned in the results, which directly outputs $k_{eff}$ and nodal power, follows a different architecture and is independently optimized with distinct hyperparameters compared to the LF + ML model. This differentiation allows for tailored optimization to suit the specific objectives of each model.

## Data Availability

The datasets used and/or analyzed during the current study are available from the corresponding author upon reasonable request.
